# MAIT cells are activated in acute Dengue virus infection and after *in vitro* Zika virus infection

**DOI:** 10.1371/journal.pntd.0006154

**Published:** 2018-01-22

**Authors:** Dominic Paquin-Proulx, Vivian I. Avelino-Silva, Bianca A. N. Santos, Nathália Silveira Barsotti, Fabiana Siroma, Jessica Fernandes Ramos, Adriana Coracini Tonacio, Alice Song, Alvino Maestri, Natalia Barros Cerqueira, Alvina Clara Felix, José Eduardo Levi, Benjamin C. Greenspun, Miguel de Mulder Rougvie, Michael G. Rosenberg, Douglas F. Nixon, Esper G. Kallas

**Affiliations:** 1 Department of Microbiology, Immunology & Tropical Medicine, The George Washington University, Washington, DC, United States of America; 2 School of Medicine, University of São Paulo, São Paulo, Brazil; 3 Hospital Sírio Libanés, São Paulo, Brazil; 4 Departamento de Molestias Infecciosas e Parasitarias-(LIM-52), Instituto de Medicina Tropical de São Paulo e Faculdade de Medicina, Universidade de São Paulo, São Paulo, Brazil; 5 Pediatric Infectious Diseases Department, Jacobi Medical Center, Bronx, NY, United States of America; Oxford University Clinical Research Unit, VIET NAM

## Abstract

Dengue virus (DENV) and Zika virus (ZIKV) are members of the *Flaviviridae* and are predominantly transmitted via mosquito bites. Both viruses are responsible for a growing number of infections in tropical and subtropical regions. DENV infection can cause lethargy with severe morbidity and dengue shock syndrome leading to death in some cases. ZIKV is now linked with Guillain-Barré syndrome and fetal malformations including microcephaly and developmental disorders (congenital Zika syndrome). The protective and pathogenic roles played by the immune response in these infections is unknown. Mucosal-associated invariant T (MAIT) cells are a population of innate T cells with potent anti-bacterial activity. MAIT cells have also been postulated to play a role in the immune response to viral infections. In this study, we evaluated MAIT cell frequency, phenotype, and function in samples from subjects with acute and convalescent DENV infection. We found that in acute DENV infection, MAIT cells had elevated co-expression of the activation markers CD38 and HLA-DR and had a poor IFNγ response following bacterial stimulation. Furthermore, we found that MAIT cells can produce IFNγ in response to *in vitro* infection with ZIKV. This MAIT cell response was independent of MR1, but dependent on IL-12 and IL-18. Our results suggest that MAIT cells may play an important role in the immune response to *Flavivirus* infections.

## Author summary

Dengue virus (DENV) and Zika virus (ZIKV) are responsible for a growing number of infections in tropical and subtropical regions. DENV infection can cause dengue shock syndrome leading to death in some cases, while ZIKV is now linked with Guillain-Barré syndrome and congenital anomalies including microcephaly. The protective and pathogenic roles played by the immune response in these infection is unknown. Mucosal-associated invariant T (MAIT) cells are a population of innate T cells with potent anti-bacterial activity. MAIT cells have also been postulated to play a role in the immune response to viral infections. In this study, we found that MAIT cells are activated in acute DENV infection and *in vitro* following ZIKV infection. MAIT cell IFNγ response to ZIKV infection was TCR independent, but IL-12 and IL-18 dependent. IFNγ produced from MAIT cells could help limit viral replication. Further studies are needed to determine the protective or pathogenic role of MAIT cells in *Flavivirus* infections.

## Introduction

Dengue virus (DENV) and Zika virus (ZIKV) are members of *Flaviviridae* and both are transmitted mostly via mosquito bites. It is estimated that around 400 million people are infected with DENV annually[1]. DENV infection symptoms range from mild disease, to dengue fever, dengue hemorrhagic fever, and dengue shock syndromes, which can be fatal in some cases. The mechanisms by which DENV infection causes severe illness are not completely understood. An extensive immune activation, characterized by a cytokine storm, has been described in DENV infection, and host factors are also likely to be involved[2]. Conventional antiviral CD8+ T cells are activated and expanded following DENV infection[3], and have been proposed to be protective by reducing the viral load[4].

Until recently, ZIKV had been understudied because the infection was thought to be associated only with a mild viral illness and of limited geographical distribution. In 2014, the virus suddenly expanded its range dramatically and appeared in the Americas, leading to the most widespread ZIKV outbreak in history. It is now estimated that over 2 billion people are living in regions suitable for ZIKV transmission[5]. ZIKV infection is now linked with cases of Guillain-Barré syndrome[6] and with a plethora of fetal malformations including microcephaly, now called congenital Zika syndrome, following transmission from an infected pregnant woman to her developing fetus[7]. The protective or pathogenic roles of T cells in ZIKV infection remains to be investigated.

Mucosal-associated invariant T (MAIT) cells are a population of innate T cells that represent 1–10% of T cells in the blood of healthy individuals[8]. They express a semi-invariant TCR using Vα7.2 coupled with Jα33 and a limited Vβ repertoire[9]. A small fraction of MAIT cells have been found to express Vα12 or Vα20[10]. Recent studies suggest that the TCR β-chain has some influence on TCR dependent activation of MAIT cells[11, 12]. MAIT cells can be identified by the expression of Vα7.2 in combination with CD161 or the IL-18 receptor[13]. They have been shown to recognize microbial vitamin B2 (riboflavin) metabolites presented by the MHC class I-like protein MR1[14]. This allows MAIT cells to respond to a range of bacteria, mycobacteria, and yeasts[15]. MAIT cells can also be activated in a TCR independent way by IL-12 and IL18[16], allowing them to respond to pathogens not producing riboflavin, such as viruses[17, 18]. In chronic HIV-1 and HTLV-1 infections, MAIT cells are reduced in number and display impaired functionality in response to bacterial stimulation[19–21]. A similar MAIT cell impairement has been described in patients with chronic infections due to a primary immunodeficiency[22].

In this study, we investigated MAIT cells response in *Flavivirus* infection. We report that MAIT cells are activated in acute DENV infection and have a poor response to *in vitro* bacterial stimulation. We also report that MAIT cells can produce IFNγ in response to *in vitro* ZIKV infection. This response was dependent on IL-12 and IL-18 and was impaired in HIV-1-infected individuals.

## Materials and methods

### Ethics statement

25 DENV-infected individuals from Sao Paulo, Brazil, were enrolled in the study (9 males and 16 females, age 17 to 87, Table 1). Patients were diagnosed with DENV infection by detection of DENV NS1 antigen and/or IgM-specific antibodies using a commercially available rapid test (Dengue Duo Test Bioeasy, Standard Diagnostic Inc. 575–34, Korea) or by detection of DENV RNA by real time PCR (RT-PCR). Absolute cell counts were determined using an automated hematology analyzer (Abbott Cell-Dyn 3700 Hematology Analyzer) at the Hematology Laboratory, Hematology Service, at the Faculty of Medicine, University of Sao Paulo. The study was approved by the University of Sao Paulo institutional review board (CAPPesq), and written informed consent was provided by all participants according to the Declaration of Helsinki. Buffy coats from healthy donors were obtained from the New York Blood Bank as approved by the George Washington University institutional review board. Samples from HIV-1-infected patients were obtained from the Jacobi Medical Center (NY, USA) and written informed consent was provided by all participants. This study was approved by Jacobi Medical Center and the George Washington University institutional review boards. All samples from all sites were anonymized. Minors were enrolled in the study, in which case legal guardians provided written informed consent according to the Declaration of Helsinki.

**Table 1:** DENV patient demographics and cell count.

DENV subject	Gender	Age	Leucocytes (cells/μL)		Neutrophiles (cells/μL)		Lymphocytes (cells/μL)		Monocytes (cells/μL)	
			Acute	Convalescent	Acute	Convalescent	Acute	Convalescent	Acute	Convalescent
1	M	28	3500	4800	1120	29820	1750	1200	460	380
2	F	25	2330	2300	700	3380	1170	2520	300	130
3	M	36	ND	ND	ND	ND	ND	ND	ND	ND
4	M	36	4640	6470	1810	3170	1900	2650	460	580
5	M	30	3280	7060	1020	3530	1640	2540	390	490
6	F	41	4080	8430	1960	5050	1630	2440	450	590
7	F	28	4280	7460	2090	4700	1670	1940	430	600
8	F	31	5610	8850	3870	6990	1180	1240	450	620
9	F	33	6180	5910	3710	5820	1790	2030	250	700
10	F	38	2460	7750	950	2840	1230	2190	150	710
11	M	59	5930	5290	2190	3640	2610	2940	800	1010
12	F	63	4510	6700	500	2060	3340	2330	360	580
13	F	64	4909	5950	1350	3750	2540	1880	900	940
14	F	17	4260	7020	1830	2800	2000	2680	380	360
15	F	35	2450	7210	830	3090	980	3090	510	700
16	M	87	4880	11840	3270	4830	680	1370	930	940
17	F	17	5570	7150	2510	8290	2010	1780	1000	1070
18	M	21	7310	4020	5410	4360	800	2430	1100	290
19	F	63	3130	8000	2160	2060	310	2330	630	580
20	F	76	2480	5630	1530	2490	600	1170	320	320
21	F	36	2700	8030	1270	4240	920	3120	490	480
22	M	47	3820	5630	2410	2540	1150	2310	530	560
23	F	42	2710	8030	1080	5380	1270	2090	250	480
24	F	66	4110	9300	2670	5670	820	2980	580	470
25	M	40	4030	7940	1890	5960	1590	1590	480	240

ND: not determined. Normal range (cells/μl); Leukocytes: 4000–11000; Neutrophils 1600–7000; Lymphocytes: 900–3400; Monocytes 200–900.

### Sample collection

Peripheral blood mononuclear cells (PBMCs) were isolated by density-gradient sedimentation using Ficoll-Paque (Lymphoprep, Nycomed Pharma, Oslo, Norway). Isolated PBMCs were washed twice in Hank’s balanced salt solution (Gibco, Grand Island, NY), and cryopreserved in RPMI 1640 (Gibco), supplemented with 20% heat inactivated fetal bovine serum (FBS; Hyclone Laboratories, Logan UT), 50 U/ml of penicillin (Gibco), 50 μg/ml of streptomycin (Gibco), 10 mM glutamine (Gibco) and 7.5% dimethylsulphoxide (DMSO; Sigma, St Louis, MO). Cryopreserved cells from all subjects were stored in liquid nitrogen until used in the assays. For DENV-infected patients, samples were collected during the acute phase of infection (before defervescence) and 1 month after (convalescent phase). Plasma was collected by centrifugation and stored at -80°C until used in the assays.

### ZIKV stock preparation

Vero cells were obtained from the American Type Culture Collection (ATCC, Manassas, VA, USA) and maintained using Eagle’s Minimum Essential Medium (ATCC) supplemented with 10% fetal bovine serum (FBS) at 37°C with 5% CO_2_. ZIKV MR766 (ATCC) was added to Vero cells at a MOI of 0.1 and incubated for 4–6 days. The supernatant was centrifuged at 12 000*g* for 5 min, filtered (0.44 μm), aliquoted and stored at –80°C. The viral titer was determined using plaque assays on Vero cells as previously described[23]. Briefly, virus stocks were serially diluted and adsorbed to confluent monolayers. After 1 h, the inoculum was removed and cells were overlaid with semisolid medium containing 1% carboxymethyl cellulose (Sigma Aldrich, St-Louis, MO, USA). Cells were further incubated for 5 days, fixed in 4% formaldehyde (Sigma Aldrich), and stained with 1% crystal violet in 20% ethanol (Sigma Aldrich) for plaque visualization. Titers were expressed as plaque forming units (PFU) per milliliter. In some experiments, ZIKV was heat inactivated by a 60 minutes incubation at 56°C.

### Flow cytometry and mAbs

Cryopreserved specimens were thawed and washed, and counts and viability were assessed using the Countess Automated Cell Counter system (Invitrogen, Carlsbad, CA). Cells were washed and stained in Brilliant Violet Stain Buffer (BD Biosciences, San Jose, CA) at room temperature for 15 min in 96-well V-bottom plates in the dark. Samples were then washed and fixed using Cytofix/Cytoperm (BD Biosciences) before flow cytometry data acquisition. Intracellular staining was performed in Perm/Wash (BD Biosciences). mAbs used in flow cytometry: CD3 AF700, CD3 PerCP-Cy5.5 (both clone UCHT1), CD8 BV711 (clone RPA-T8), CD38 APC-H7 (clone HB7), CD127 FITC (clone HIL-7R-M2), CD161 BV421 (clone DX12), CCR6 BV786 (clone 11A9), HLA-DR APC (clone L243), IFNγ APC (clone B27), and PD-1 PE-Cy7 (clone EH12.1) were all from BD Biosciences, PLZF APC was from R&D Systems (Minneapolis, MN), EOMES FITC (clone WD1928) was from eBioscience and TCR Vα7.2 PE (clone 3C10) was from Biolegend (San Diego, CA, USA). Live/dead aqua fixable cell stain was from Life Technologies (Eugene, OR, USA). Data were acquired on a BD LSRFortessa instrument (BD Biosciences) and analyzed using FlowJo Version 9.8.5 software (TreeStar, Ashland, OR, USA).

### Functional assay

MAIT cell function was determined *in vitro* using paraformaldehyde-fixed *E*. *coli* stimulation (one shot top10, Life Technology, multiplicity of exposure 10) in the presence of 1.25 μg/ml anti-CD28 mAb (clone L293, BD Biosciences)[24] or ZIKV at a MOI of 5 (without anti CD28 mAb). *E*. *coli* was fixed for 5 minutes in 1% paraformaldehyde. PBMCs were further cultured for 24 hours at 37°C/5% CO_2_ in RPMI medium supplemented with 10% fetal bovin serum. Monensin (Golgi Stop, BD Biosciences) was added during the last 6 hours of the stimulation. In some experiments blocking antibodies against MR-1 (5μg/ml, clone 26.5, Biolegend), IL-12p70 (10μg/ml, clone 24910, R&D systems), and IL-18 (10μg/ml, clone 125-2H, MBL International, Woburn, MA, USA) were added.

### IL-7 and soluble CD14 (sCD14) measurement

IL-7 (RayBiotech, Norcross, GA, USA) and sCD14 (R&D Systems) were measured in plasma by ELISA following manufacturer’s instruction.

### Statistical analysis

All statistical analysis was performed using Graph Pad Prism version 6.0h for Mac OSX (GraphPad Software, La Jolla, CA). The changes between acute and convalescent phases and before/after ZIKV stimulation with or without blocking antibodies were analyzed with Wilcoxon matched-pairs signed rank test. Associations between groups were determined by Spearman's rank correlation. P-values ≤ 0.05 were considered statistically significant.

## Results

**Fig 1:**
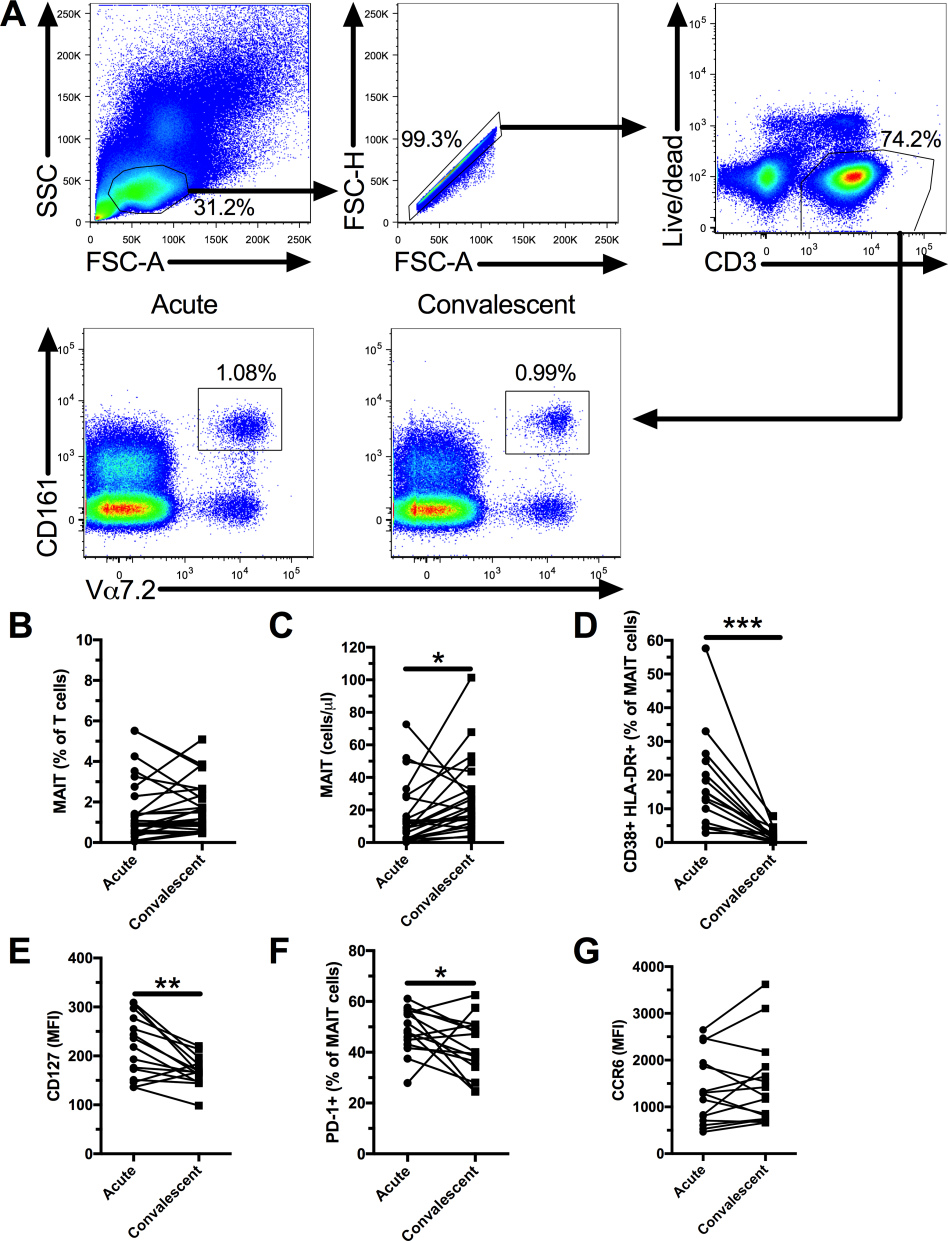
MAIT cells have elevated expression of activation markers in acute DENV infection. Gating strategy and representative flow plots of MAIT cell frequency (A). MAIT cell frequency in acute and convalescent DENV infection (n = 25, B). MAIT cell count, (as determined by multiplying the lymphocyte count by the frequency of MAIT cells in lymphocytes), in acute and convalescent DENV infection (n = 24, C). Co-expression of CD38 and HLA-DR by MAIT cells in acute and convalescent DENV infection (n = 15, D). CD127 expression (MFI) by MAIT cells in acute and convalescent DENV infection (n = 15, E). PD-1 expression by MAIT cells in acute and convalescent DENV infection (n = 15, F). CCR6 expression (MFI) by MAIT cells in acute and convalescent DENV infection (n = 15, G). ** indicates p < 0.01, and *** indicates p < 0.001.

We enrolled 25 individuals with acute DENV infection, and we followed them during the convalescent phase (Table 1). We evaluated MAIT cell (defined as CD3+ CD161+ Vα7.2+, Fig 1A) frequency by flow cytometry and found no significant difference between acute and convalescent DENV infection (Fig 1B). However, MAIT cell counts were decreased in the acute phase (Fig 1C) due to significant overall lymphopenia amongst infected patients (S1 Fig). Next, we characterized the phenotype of MAIT cells in the acute and convalescent phases of DENV infection. MAIT cells had significantly increased co-expression of the activation markers CD38 and HLA-DR (S1 Fig and Fig 1D), of the IL-7 receptor CD127 (S1 Fig and Fig 1E), and of PD-1 (S1 Fig and Fig 1F) in the acute phase. We did not observe any difference in the expression of CCR6 by MAIT between the acute and convalescent phases (Fig 1G). In chronic viral infections MAIT cell activation is associated with their reduced frequency[19, 21]. Thus, we investigated if there was an association between the reduced MAIT cell count in the acute phase and their increased co-expression of CD38 and HLA-DR, and found a trend for an inverse association (p = 0.0779, S1 Fig). Next, we compared the results for MAIT cells during the convalescent phase to healthy controls from Brazil. We found that there was no difference in the co-expression of CD38 and HLA-DR between the convalescent and healthy controls individuals (S2 Fig). PD-1 remained elevated during the convalescent phase of DENV infection (S2 Fig) and CD127 was decreased compared to healthy controls (S2 Fig). Our results show that MAIT cells are activated and reduced in number in acute DENV infection.

Because the majority of MAIT cells are CD8+, we evaluated the response of conventional CD8 T cells in acute DENV infection. Conventional CD8 T cells had significantly elevated levels of co-expression of CD38 and HLA-DR in the acute phase and the levels of co-expression in the convalescent phase were similar to healthy controls (S3 Fig). PD-1 was also elevated on CD8 T cell in the acute phase of infection. However, PD-1 levels in the convalescent phase trend to remain elevated compared to healthy controls (S3 Fig). However, in contrast to MAIT cells, the levels of CD127 on conventional CD8 T cells were not different between the acute and the convalescent phase, or healthy controls (S3 Fig).

**Fig 2:**
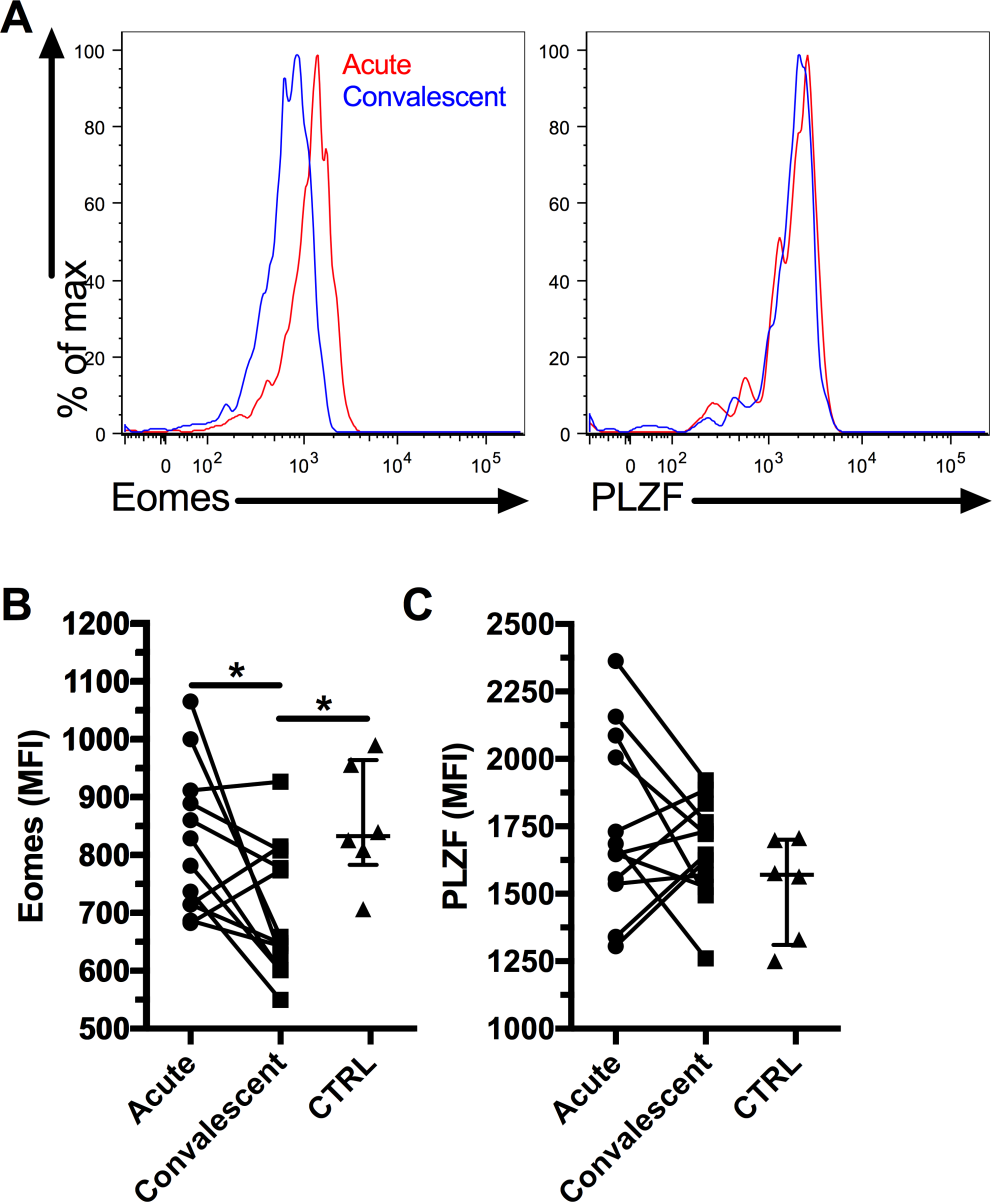
Reduced Eomes expression by MAIT cells in convalescent DENV infection. Representative flow plots for Eomes and PLZF expression by MAIT cells (A). Eomes (B) and PLZF (C) expression (MFI) by MAIT cells in acute and convalescent DENV infection (n = 12) and healthy control (n = 6). The bars and whiskers represent the median and interquartile range respectively. * indicates p < 0.05.

MAIT cells have been shown to have decreased expression of key transcription factors in chronic viral infections[21, 25, 26]. Therefore, we investigated if MAIT cells showed a similar decrease of Eomes and PLZF expression in acute DENV infection. We found that Eomes expression was reduced in convalescent DENV infection (Fig 2A and 2B) compared to the acute phase and healthy controls. However, we did not observe any difference in PLZF expression between acute and convalescent DENV infection or healthy controls (Fig 2A and 2C). Our results suggest that different transcription factor expression profiles are associated with acute and chronic viral infections respectively.

**Fig 3:**
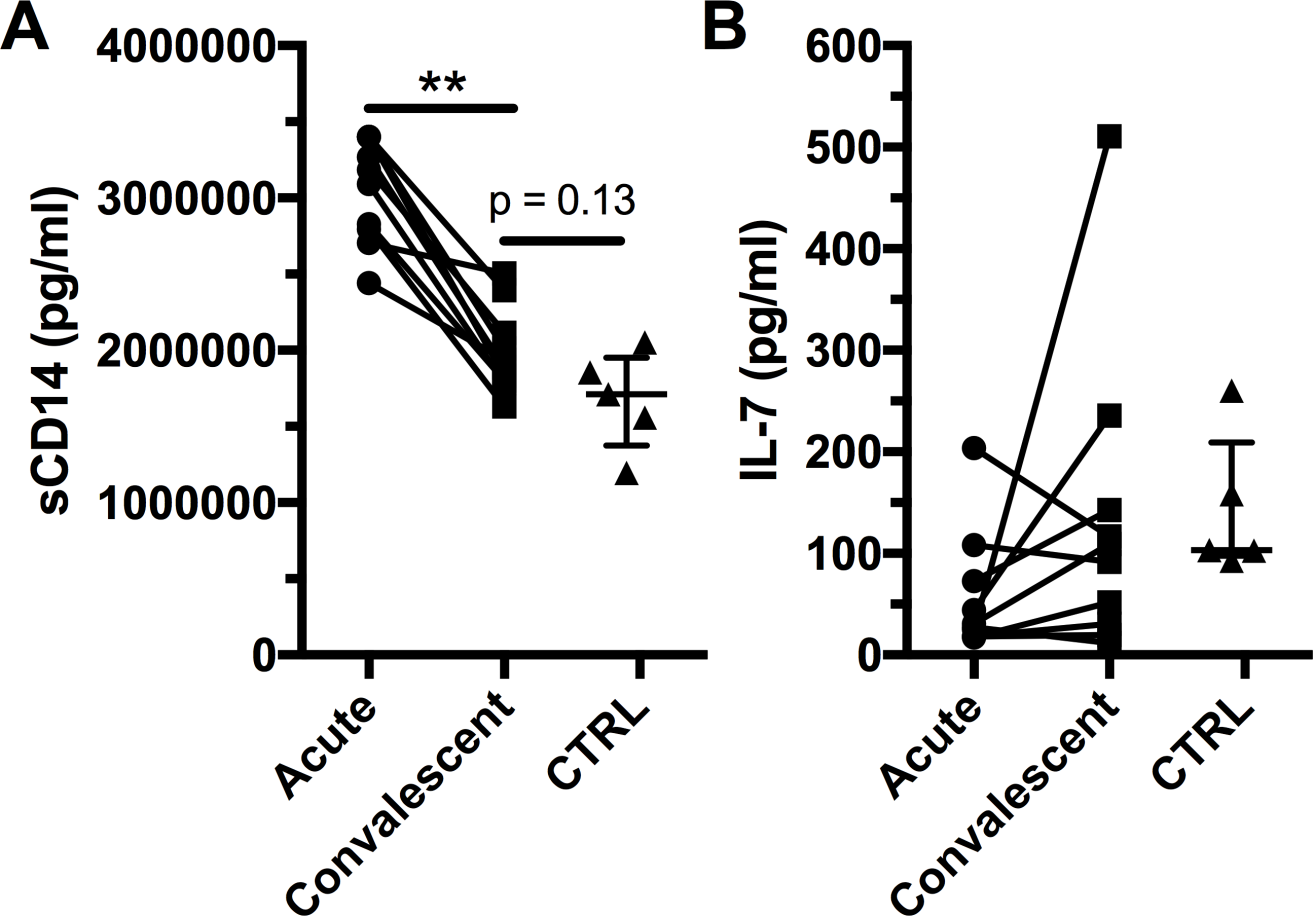
Increased levels of sCD14 in acute DENV infection. Plasma levels of sCD14 (A) and IL-7 (B) in acute and convalescent DENV infection were determined for subjects 1–10 (n = 10) and for healthy control (n = 5). The bars and whiskers represent the median and interquartile range respectively. ** indicates p < 0.01.

Increased pro-inflammatory cytokines levels in DENV infection have been associated with microbial translocation[27]. sCD14 is a marker of monocyte activation and is considered an indirect marker of microbial translocation[28]. Thus, we measured the levels of sCD14 in our cohort of DENV-infected subjects. Levels of sCD14 were significantly higher in the acute phase of infection than in the convalescent phase (Fig 3A). Levels of sCD14 remained higher in convalescent DENV compared to healthy controls. However, we did not find any significant associations between the levels of sCD14 in acute DENV infection and co-expression of CD38 and HLA-DR by MAIT cells or with MAIT cell numbers (S4 Fig). Because we found elevated expression of the IL-7 receptor by MAIT cells in acute DENV infection, we measured the levels of plasma IL-7 in acute and convalescent DENV infection but did not find any significant change (Fig 3B).

**Fig 4:**
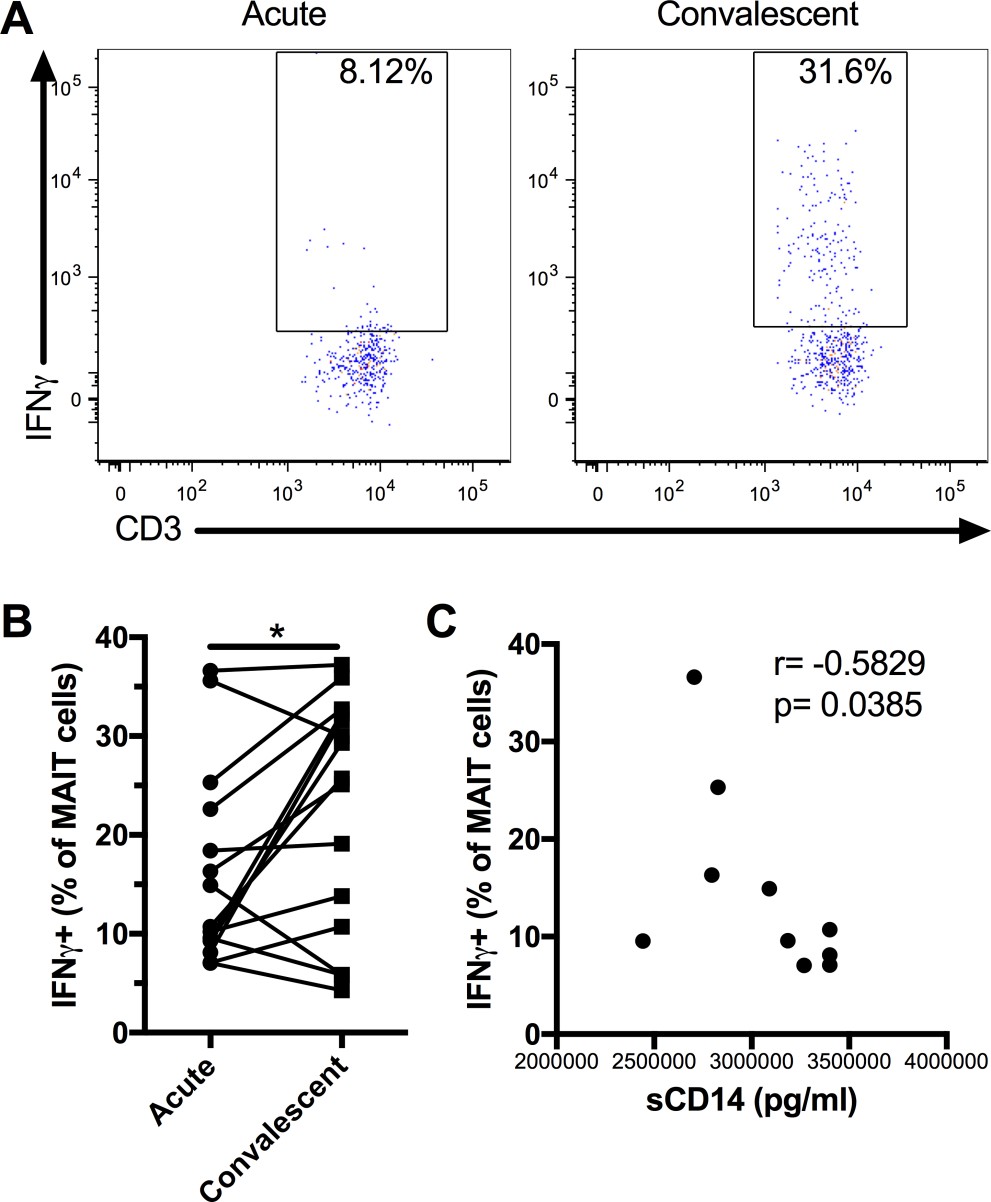
Lower IFNγ production following *in vitro* stimulation with *E*. *coli* by MAIT cells in acute DENV infection. MAIT cells were stimulated with fixed *E*. *coli* (multiplicity of exposure 10) for 24h and IFNγ production by MAIT cells was evaluated by flow cytometry. Representative flow plots of IFNγ production by MAIT cells (A). IFNγ production by MAIT cells in response to *E*. *coli* stimulation in acute and convalescent DENV infection (n = 15, B). Association between the levels of sCD14 and MAIT cells IFNγ response to *E*. *coli* stimulation in acute DENV infection (C). * indicates p < 0.05.

Next, to establish the functionality of MAIT cells, we investigated the *in vitro* response of MAIT cells from the acute and convalescent phases of DENV infection to *in vitro* stimulation with *E*. *coli*. There was no difference in IFNγ production by MAIT cells in the acute and convalescent phases of DENV infection in the absence of stimulation (S5 Fig). MAIT cells in the acute phase produced significantly less IFNγ after *E*. *coli* stimulation compared to the convalescent phase (Fig 4A and 4B). The MAIT cell IFNγ response in the convalescent phase was similar to the response of healthy controls (S5 Fig). Interestingly, we found that the levels of sCD14 in acute DENV infection were inversely associated with the MAIT cell IFNγ response (Fig 4C), possibly suggesting a role for monocyte activation in the poor MAIT cell response.

**Fig 5:**
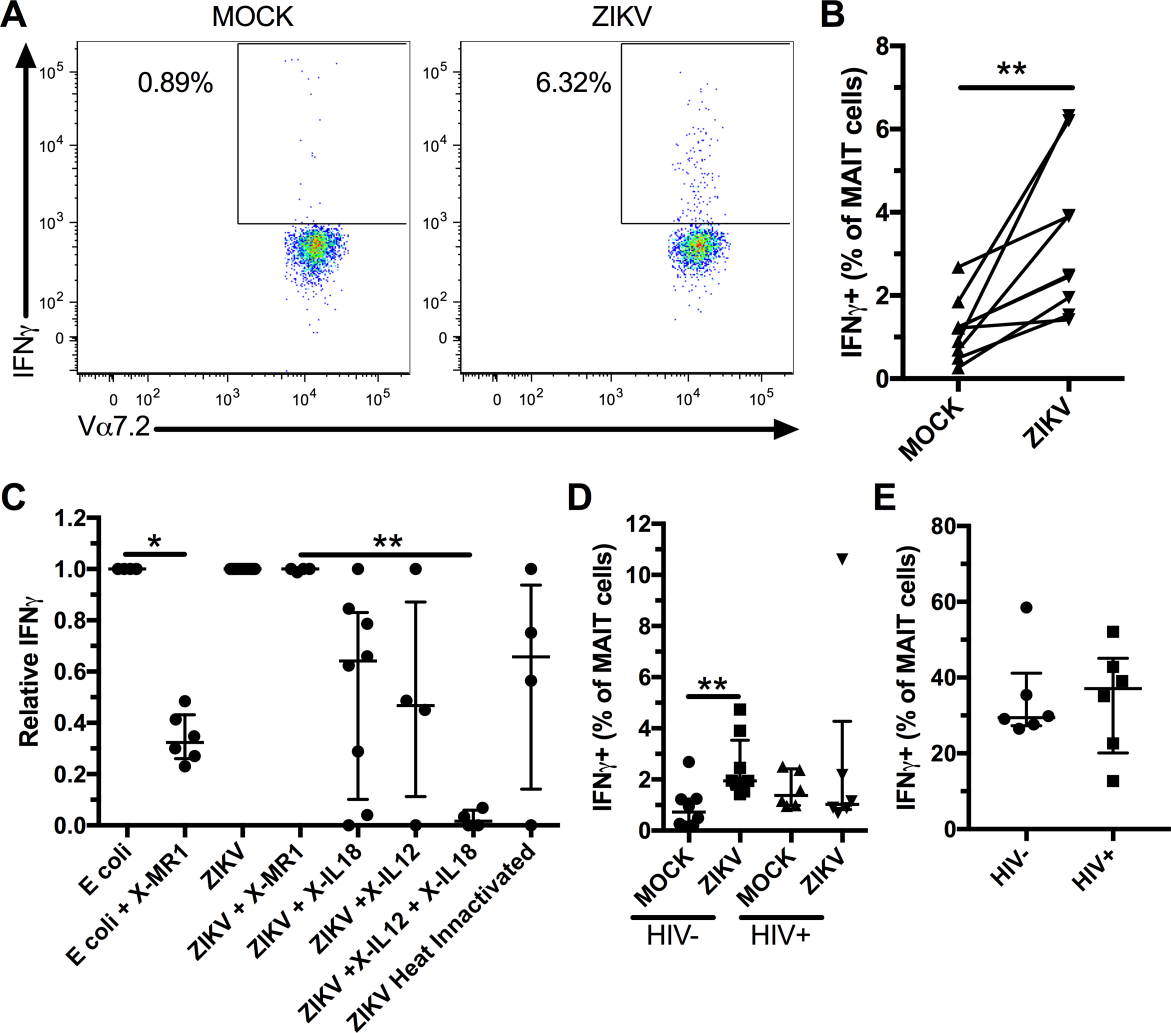
MAIT cells response to *in vitro* ZIKV infection is dependent on IL-12 and IL-18. PBMCs were infected with ZIKV (MOI: 5) for 24h and IFNγ production by MAIT cells was evaluated by flow cytometry. Representative flow plots of IFNγ production by MAIT cells (A). MAIT cells IFNγ response to ZIKV infection (n = 9, B). PBMCs were incubated in presence of MR1 (n = 6), IL-12 (n = 4), IL-18 (n = 8), and IL-12 + IL-18 (n = 4) blocking antibodies and stimulated with *E*. *coli*, ZIKV, or heat inactivated ZIKV (n = 4) and IFNγ production by MAIT cells was measured (C). The results are presented as relative production of IFNγ by MAIT cells compared to the condition without any blocking antibody. Statistical analysis was performed before data normalization. PBMCs from HIV-1- (n = 8) and HIV-1+ (n = 6) subjects were infected with ZIKV (MOI: 5) and IFNγ production by MAIT cell was evaluated (D). PBMCs from HIV-1- (n = 6) and HIV-1+ (n = 6) subjects were treated with IL-12 and IL-18 (both at 50ng/ml) for 24 hours and IFNγ production by MAIT cell was evaluated (E). The bars and whiskers represent the median and interquartile range respectively. * indicates p < 0.05 and ** indicates p < 0.01.

Finally, we used *in vitro* infection with ZIKV to study the mechanism of MAIT cell activation in a different *Flavivirus* infection. MAIT cells from healthy individuals consistently produced IFNγ in response to *in vitro* ZIKV infection (Fig 5A and 5B). In contrast to *E*. *coli*, the MAIT cell IFNγ response to ZIKV could not be blocked by a MR-1 blocking antibody (Fig 5C). The IFNγ response from MAIT cells to ZIKV was partially reduced by blocking antibodies against IL-12 and IL-18 and was completely blocked when they were used in combination (Fig 5C). We also investigated if viral replication was needed for the MAIT cell response to *in vitro* ZIKV infection. For this purpose, heat inactivated ZIKV was added to PBMCs and the MAIT cell IFNγ response was compared to the response obtained using replication competent ZIKV. We observed only a small reduction in IFNγ production by MAIT cells in response to ZIKV when using a heat inactivated virus (Fig 5C), suggesting that viral replication is not needed for production of IL-12 and IL-18 and subsequent MAIT cell activation.

Viral co-infections are common and understudied. In Brazil, many people living with HIV will be exposed to dengue or zika viruses. MAIT cells from HIV-1-infected individuals exhibit decreased functionality following stimulation with *E*. *coli*[19]. Thus, we evaluated the capacity of MAIT cells from HIV-1-infected subjects (Table 2) to produce IFNγ in response to ZIKV infection and found that in 5 out of 6 individuals there was no increase in IFNγ production in response to ZIKV infection (Fig 5D). We then directly stimulated PBMCs with IL-12 and IL-18 and found a similar IFNγ response by MAIT cells from HIV-1-infected and uninfected subjects (Fig 5E), suggesting that MAIT cells from HIV-1-infected individuals have a normal capacity to respond to cytokine stimulation.

**Table 2:** HIV-1 patients demographics and characteristics.

HIV Subject	Gender	Age	Treatment	Comment
1	M	15.2	Yes	
2	F	17.2	Yes	Variable treatment adherence
3	F	17.8	Yes	
4	M	26.8	Yes	
5	M	25.0	No	Non progressor
6	M	18.8	Yes	

## Discussion

We found that MAIT cells are activated in acute human DENV infection as well as following *in vitro* ZIKV infection. However, in contrast to a previous study[17], we did not find any significant changes in MAIT cell frequency between acute and convalescent DENV infection. Wilgenburg *et al*. focused on the study of CD8+ MAIT cells, while in this study, we also included CD8- MAIT cells. In addition, the difference in timing of sample collection might explain the differences between the two studies. We found that MAIT cell counts were decreased in parallel with the total lymphocyte count during the acute phase. We found that MAIT cells were activated during acute DENV infection, as had Wilgenburg and colleagues. IL-12 and IL-18 have been shown to trigger MAIT cell activation[16], and monocyte production of IL-18 is required for MAIT cell *in vitro* response to influenza A virus (IAV)[18]. The levels of IL-12 and IL-18 are elevated in DENV infection[17, 29–31] and could therefore be involved in MAIT cell activation, as shown here. We showed that MAIT cell IFNγ production following *in vitro* ZIKV infection also depended on IL-12 and IL-18. Immune activation in acute DENV infection has been associated with elevated levels of LPS and of markers of microbial translocation[27, 32]. This raises the possibility that MAIT cells could also be activated in a TCR-dependent way by microbial products during acute DENV. However, we did not find an association between the levels of sCD14, an indirect marker of microbial translocation, and MAIT cell activation in acute DENV. Rather, we found that sCD14 was inversely associated with the *in vitro* IFNγ response of MAIT cells to *E*. *coli*. This suggests that monocyte activation could result in poor antigen presentation to MAIT cells. An alternative explanation could be of a temporary monocyte tolerance to stimulation induced by LPS. This could contribute to the reduced MAIT cell response in the acute phase. Finally, the elevated expression of PD-1 on MAIT cells during acute DENV infection could also contribute to the reduced IFNγ production. Further studies are needed to confirm that both MAIT cells and monocytes are involved in this defect.

Chronic viral infections have been associated with a reduced expression of the transcription factors PLZF and Eomes by MAIT cells[21, 25, 26]. Interestingly, we found that DENV infection did not change the levels of PLZF expression in MAIT cells and their Eomes levels were reduced in convalescent compared to acute DENV and healthy controls. CD56+ MAIT cells have been shown to have a higher Eomes expression and a more robust response to IL-12 and IL-18 than CD56- MAIT cells [11]. Therefore, it is possible that the decrease in Eomes expression by MAIT cells in convalescent DENV infection is part of a feedback loop to decrease their response to cytokines. Another possibility is a decrease in the CD56+ subset of MAIT cells in blood following acute DENV. We have also observed a decreased expression of the IL-7 receptor (CD127) by MAIT cells during the convalescent phase. IL-7 has been shown to increase MAIT cell response[26, 33]. Thus, reduced levels of Eomes and CD127 could by a mechanism by which MAIT cells could prevent sustained activation following an acute infection.

Patients that recovered from IAV infection had higher circulating MAIT cells than those that succumbed[18] and IFNγ production by MAIT cells has been shown to limit HCV replication *in vitro[17]*. Thus, there is increasing evidence that MAIT cells could play a protective role in viral infections. DENV and ZIKV infections are associated with a range of clinical symptoms. More studies are needed to investigate if MAIT cell frequency, functionality or activation status have an impact on the clinical outcome of DENV and ZIKV infections. In this regard, MAIT cell production of IFNγ could be part of an innate immune response to induce an anti-viral state and compromise *Flavivirus* replication. Levels of serum IFNγ have been reported to be inversely associated with DENV load and symptoms[34]. One limitation of our study is that we focused only on peripheral MAIT cells. MAIT cells are present in the skin[35, 36] and skin resident MAIT cells may play a more important role in early innate defense following mosquito transmission of *Flavivirus*.

MAIT cells from HIV-1-infected individuals have been shown to have a lower production of cytokines in response to *E*. *coli* stimulation[19]. In this study, we show that the cytokine mediated MAIT cell response to *in vitro* viral infection is also impaired. However, MAIT cells from HIV-1-infected subjects had a normal response to direct cytokine stimulation, suggesting that poor IL-12 and IL-18 production in response to ZIKV infection could be responsible for the impaired MAIT cell response in these individuals. This suggests that HIV-1-infected individuals could have a poor innate immune response to ZIKV and be at a higher risk to develop complications following *Flavivirus* infection. Case reports of HIV-1-infected individuals with ZIKV infection have been reported[37, 38], including one case with congenital Zika syndrome[39]. Defective MAIT cell activation could be one factor contributing to the increase incidence of severe dengue in HIV-1-infected subjects[40]. More studies are needed to determine if MAIT cells contribute to protection or to immunopathology during *Flavivirus* infections.

Overall, our results show that MAIT cells are activated in response to DENV and ZIKV infections. This innate response was TCR-independent and defective in HIV-1-infected individuals. Further studies are necessary to determine the importance of MAIT cell responses in the clinical outcomes of *Flavivirus* infections.

## Supporting information

S1 Fig Lymphocytes count in acute and convalescent DENV infection (n = 24, A). Representative flow plots of CD38 and HLA-DR co-expression by MAIT cells in acute and convalescent DENV infection (B). CD127 expression level by MAIT cells in acute and convalescent DENV infection (C). Representative flow plots of PD-1 expression by MAIT cells in acute and convalescent DENV infection (D). Association between CD38 and HLA-DR co-expression and MAIT cells count in acute DENV infection (E).
(TIFF)

S2 Fig MAIT cell frequency in convalescent DENV infection (n = 25) and healthy controls (n = 26), (A). Co-expression of CD38 and HLA-DR by MAIT cells in convalescent DENV infection (n = 15) and healthy controls (n = 26), (B). PD-1 expression by MAIT cells in convalescent DENV infection (n = 15) and healthy controls (n = 26), (C). CD127 expression (MFI) by MAIT cells in convalescent DENV infection (n = 15) and healthy controls (n = 7), (D). * indicates p < 0.05, and ** indicates p < 0.01.
(TIFF)

S3 Fig Co-expression of CD38 and HLA-DR by conventional CD8 T cells (excluding MAIT cells) during acute and convalescent DENV infection (n = 10) and healthy controls (n = 23) (A). PD-1 expression by conventional CD8 T cells in acute and convalescent DENV infection (n = 10) and healthy controls (n = 23) (B). CD127 expression by convetional CD8 T cells in acute and convalescent DENV infection (n = 10) and healthy controls (n = 5) (C). The bars and whiskers represent the median and interquartile range, respectively. * indicates p < 0.05 and ** indicates p < 0.01.
(TIFF)

S4 Fig Associations between sCD14 levels and co-expression of CD38 and HLA-DR by MAIT cells (A) and MAIT cell count (B) in acute DENV infection.
(TIFF)

S5 Fig Representative flow plots showing IFNγ production by unstimulated MAIT cells in acute and convalescent dengue infection (A). IFNγ production by unstimulated MAIT cells in acute and convalescent dengue infection (n = 12, B). IFNγ production by *E*. *coli* stimulated MAIT cells in convalescent dengue infection (n = 15) and control subjects (n = 10) (C). The bars and whiskers represent the median and interquartile range, respectively.
(TIFF)

## References

[1] Bhatt S, Gething PW, Brady OJ, Messina JP, Farlow AW, Moyes CL, et al The global distribution and burden of dengue. Nature. 2013;496(7446):504–7. doi: 10.1038/nature12060 ; PubMed Central PMCID: PMCPMC3651993. 23563266 10.1038/nature12060 PMC3651993

[2] Rothman AL. Immunity to dengue virus: a tale of original antigenic sin and tropical cytokine storms. Nat Rev Immunol. 2011;11(8):532–43. doi: 10.1038/nri3014 . 21760609 10.1038/nri3014

[3] de Matos AM, Carvalho KI, Rosa DS, Villas-Boas LS, da Silva WC, Rodrigues CL, et al CD8+ T lymphocyte expansion, proliferation and activation in dengue fever. PLoS Negl Trop Dis. 2015;9(2):e0003520 doi: 10.1371/journal.pntd.0003520 ; PubMed Central PMCID: PMCPMC4326415. 25675375 10.1371/journal.pntd.0003520 PMC4326415

[4] Yauch LE, Zellweger RM, Kotturi MF, Qutubuddin A, Sidney J, Peters B, et al A protective role for dengue virus-specific CD8+ T cells. J Immunol. 2009;182(8):4865–73. doi: 10.4049/jimmunol.0801974 ; PubMed Central PMCID: PMCPMC2674070. 19342665 10.4049/jimmunol.0801974 PMC2674070

[5] Messina JP, Kraemer MU, Brady OJ, Pigott DM, Shearer FM, Weiss DJ, et al Mapping global environmental suitability for Zika virus. Elife. 2016;5 doi: 10.7554/eLife.15272 ; PubMed Central PMCID: PMCPMC4889326. 27090089 10.7554/eLife.15272 PMC4889326

[6] Cao-Lormeau VM, Blake A, Mons S, Lastere S, Roche C, Vanhomwegen J, et al Guillain-Barre Syndrome outbreak associated with Zika virus infection in French Polynesia: a case-control study. Lancet. 2016;387(10027):1531–9. doi: 10.1016/S0140-6736(16)00562-6 ; PubMed Central PMCID: PMCPMC5444521. 26948433 10.1016/S0140-6736(16)00562-6 PMC5444521

[7] Rasmussen SA, Jamieson DJ, Honein MA, Petersen LR. Zika Virus and Birth Defects—Reviewing the Evidence for Causality. N Engl J Med. 2016;374(20):1981–7. doi: 10.1056/NEJMsr1604338 . 27074377 10.1056/NEJMsr1604338

[8] Salou M, Franciszkiewicz K, Lantz O. MAIT cells in infectious diseases. Curr Opin Immunol. 2017;48:7–14. doi: 10.1016/j.coi.2017.07.009 . 28750261 10.1016/j.coi.2017.07.009

[9] Treiner E, Duban L, Bahram S, Radosavljevic M, Wanner V, Tilloy F, et al Selection of evolutionarily conserved mucosal-associated invariant T cells by MR1. Nature. 2003;422(6928):164–9. doi: 10.1038/nature01433 . 12634786 10.1038/nature01433

[10] Reantragoon R, Corbett AJ, Sakala IG, Gherardin NA, Furness JB, Chen Z, et al Antigen-loaded MR1 tetramers define T cell receptor heterogeneity in mucosal-associated invariant T cells. J Exp Med. 2013;210(11):2305–20. doi: 10.1084/jem.20130958 ; PubMed Central PMCID: PMCPMC3804952. 24101382 10.1084/jem.20130958 PMC3804952

[11] Dias J, Leeansyah E, Sandberg JK. Multiple layers of heterogeneity and subset diversity in human MAIT cell responses to distinct microorganisms and to innate cytokines. Proc Natl Acad Sci U S A. 2017 doi: 10.1073/pnas.1705759114 . 28630305 10.1073/pnas.1705759114 PMC5502643

[12] Gold MC, McLaren JE, Reistetter JA, Smyk-Pearson S, Ladell K, Swarbrick GM, et al MR1-restricted MAIT cells display ligand discrimination and pathogen selectivity through distinct T cell receptor usage. J Exp Med. 2014;211(8):1601–10. doi: 10.1084/jem.20140507 ; PubMed Central PMCID: PMCPMC4113934. 25049333 10.1084/jem.20140507 PMC4113934

[13] Leeansyah E, Loh L, Nixon DF, Sandberg JK. Acquisition of innate-like microbial reactivity in mucosal tissues during human fetal MAIT-cell development. Nat Commun. 2014;5:3143 doi: 10.1038/ncomms4143 ; PubMed Central PMCID: PMCPMC3916833. 24452018 10.1038/ncomms4143 PMC3916833

[14] Kjer-Nielsen L, Patel O, Corbett AJ, Le Nours J, Meehan B, Liu L, et al MR1 presents microbial vitamin B metabolites to MAIT cells. Nature. 2012;491(7426):717–23. doi: 10.1038/nature11605 . 23051753 10.1038/nature11605

[15] Le Bourhis L, Martin E, Peguillet I, Guihot A, Froux N, Core M, et al Antimicrobial activity of mucosal-associated invariant T cells. Nat Immunol. 2010;11(8):701–8. doi: 10.1038/ni.1890 . 20581831 10.1038/ni.1890

[16] Ussher JE, Bilton M, Attwod E, Shadwell J, Richardson R, de Lara C, et al CD161++ CD8+ T cells, including the MAIT cell subset, are specifically activated by IL-12+IL-18 in a TCR-independent manner. Eur J Immunol. 2014;44(1):195–203. doi: 10.1002/eji.201343509 ; PubMed Central PMCID: PMCPMC3947164. 24019201 10.1002/eji.201343509 PMC3947164

[17] van Wilgenburg B, Scherwitzl I, Hutchinson EC, Leng T, Kurioka A, Kulicke C, et al MAIT cells are activated during human viral infections. Nat Commun. 2016;7:11653 doi: 10.1038/ncomms11653 ; PubMed Central PMCID: PMCPMC4931007. 27337592 10.1038/ncomms11653 PMC4931007

[18] Loh L, Wang Z, Sant S, Koutsakos M, Jegaskanda S, Corbett AJ, et al Human mucosal-associated invariant T cells contribute to antiviral influenza immunity via IL-18-dependent activation. Proc Natl Acad Sci U S A. 2016;113(36):10133–8. doi: 10.1073/pnas.1610750113 ; PubMed Central PMCID: PMCPMC5018778. 27543331 10.1073/pnas.1610750113 PMC5018778

[19] Leeansyah E, Ganesh A, Quigley MF, Sonnerborg A, Andersson J, Hunt PW, et al Activation, exhaustion, and persistent decline of the antimicrobial MR1-restricted MAIT-cell population in chronic HIV-1 infection. Blood. 2013;121(7):1124–35. doi: 10.1182/blood-2012-07-445429 ; PubMed Central PMCID: PMCPMC3575756. 23243281 10.1182/blood-2012-07-445429 PMC3575756

[20] Cosgrove C, Ussher JE, Rauch A, Gartner K, Kurioka A, Huhn MH, et al Early and nonreversible decrease of CD161++ /MAIT cells in HIV infection. Blood. 2013;121(6):951–61. doi: 10.1182/blood-2012-06-436436 ; PubMed Central PMCID: PMCPMC3567342. 23255555 10.1182/blood-2012-06-436436 PMC3567342

[21] Paquin-Proulx D, Greenspun BC, Costa EA, Segurado AC, Kallas EG, Nixon DF, et al MAIT cells are reduced in frequency and functionally impaired in human T lymphotropic virus type 1 infection: Potential clinical implications. PLoS One. 2017;12(4):e0175345 doi: 10.1371/journal.pone.0175345 ; PubMed Central PMCID: PMCPMC5383303. 28384290 10.1371/journal.pone.0175345 PMC5383303

[22] Paquin-Proulx D, Santos BAN, Silveira Barsotti N, Marinho AKBB, Kokron CM, Carvalho KI, et al Loss of Circulating Mucosal-Associated Invariant T Cells in Common Variable Immunodeficiency Is Associated with Immune Activation and Loss of Eomes and PLZF. ImmunoHorizon. 2017;1(7):141–55. doi: 10.4049/immunohorizons.1700039

[23] Delvecchio R, Higa LM, Pezzuto P, Valadao AL, Garcez PP, Monteiro FL, et al Chloroquine, an Endocytosis Blocking Agent, Inhibits Zika Virus Infection in Different Cell Models. Viruses. 2016;8(12). doi: 10.3390/v8120322 ; PubMed Central PMCID: PMCPMC5192383. 27916837 10.3390/v8120322 PMC5192383

[24] Dias J, Sobkowiak MJ, Sandberg JK, Leeansyah E. Human MAIT-cell responses to Escherichia coli: activation, cytokine production, proliferation, and cytotoxicity. J Leukoc Biol. 2016;100(1):233–40. doi: 10.1189/jlb.4TA0815-391RR ; PubMed Central PMCID: PMCPMC4946616. 27034405 10.1189/jlb.4TA0815-391RR PMC4946616

[25] Hengst J, Strunz B, Deterding K, Ljunggren HG, Leeansyah E, Manns MP, et al Nonreversible MAIT cell-dysfunction in chronic hepatitis C virus infection despite successful interferon-free therapy. Eur J Immunol. 2016;46(9):2204–10. doi: 10.1002/eji.201646447 . 27296288 10.1002/eji.201646447

[26] Leeansyah E, Svard J, Dias J, Buggert M, Nystrom J, Quigley MF, et al Arming of MAIT Cell Cytolytic Antimicrobial Activity Is Induced by IL-7 and Defective in HIV-1 Infection. PLoS Pathog. 2015;11(8):e1005072 doi: 10.1371/journal.ppat.1005072 ; PubMed Central PMCID: PMCPMC4546682. 26295709 10.1371/journal.ppat.1005072 PMC4546682

[27] van de Weg CA, Pannuti CS, de Araujo ES, van den Ham HJ, Andeweg AC, Boas LS, et al Microbial translocation is associated with extensive immune activation in dengue virus infected patients with severe disease. PLoS Negl Trop Dis. 2013;7(5):e2236 doi: 10.1371/journal.pntd.0002236 ; PubMed Central PMCID: PMCPMC3662706. 23717702 10.1371/journal.pntd.0002236 PMC3662706

[28] Leeansyah E, Malone DF, Anthony DD, Sandberg JK. Soluble biomarkers of HIV transmission, disease progression and comorbidities. Curr Opin HIV AIDS. 2013;8(2):117–24. doi: 10.1097/COH.0b013e32835c7134 . 23274365 10.1097/COH.0b013e32835c7134

[29] Mustafa AS, Elbishbishi EA, Agarwal R, Chaturvedi UC. Elevated levels of interleukin-13 and IL-18 in patients with dengue hemorrhagic fever. FEMS Immunol Med Microbiol. 2001;30(3):229–33. . 11335143 10.1111/j.1574-695X.2001.tb01575.x

[30] Fagundes CT, Costa VV, Cisalpino D, Amaral FA, Souza PR, Souza RS, et al IFN-gamma production depends on IL-12 and IL-18 combined action and mediates host resistance to dengue virus infection in a nitric oxide-dependent manner. PLoS Negl Trop Dis. 2011;5(12):e1449 doi: 10.1371/journal.pntd.0001449 ; PubMed Central PMCID: PMCPMC3243710. 22206036 10.1371/journal.pntd.0001449 PMC3243710

[31] Pacsa AS, Agarwal R, Elbishbishi EA, Chaturvedi UC, Nagar R, Mustafa AS. Role of interleukin-12 in patients with dengue hemorrhagic fever. FEMS Immunol Med Microbiol. 2000;28(2):151–5. . 10799806 10.1111/j.1574-695X.2000.tb01470.x

[32] van de Weg CA, Koraka P, van Gorp EC, Mairuhu AT, Supriatna M, Soemantri A, et al Lipopolysaccharide levels are elevated in dengue virus infected patients and correlate with disease severity. J Clin Virol. 2012;53(1):38–42. doi: 10.1016/j.jcv.2011.09.028 . 22014848 10.1016/j.jcv.2011.09.028

[33] Tang XZ, Jo J, Tan AT, Sandalova E, Chia A, Tan KC, et al IL-7 licenses activation of human liver intrasinusoidal mucosal-associated invariant T cells. J Immunol. 2013;190(7):3142–52. doi: 10.4049/jimmunol.1203218 . 23447689 10.4049/jimmunol.1203218

[34] Pal T, Dutta SK, Mandal S, Saha B, Tripathi A. Differential clinical symptoms among acute phase Indian patients revealed significant association with dengue viral load and serum IFN-gamma level. J Clin Virol. 2014;61(3):365–70. doi: 10.1016/j.jcv.2014.09.003 . 25288310 10.1016/j.jcv.2014.09.003

[35] Li J, Reantragoon R, Kostenko L, Corbett AJ, Varigos G, Carbone FR. The frequency of mucosal-associated invariant T cells is selectively increased in dermatitis herpetiformis. Australas J Dermatol. 2016 doi: 10.1111/ajd.12456 . 26940855 10.1111/ajd.12456

[36] Teunissen MB, Yeremenko NG, Baeten DL, Chielie S, Spuls PI, de Rie MA, et al The IL-17A-producing CD8+ T-cell population in psoriatic lesional skin comprises mucosa-associated invariant T cells and conventional T cells. J Invest Dermatol. 2014;134(12):2898–907. doi: 10.1038/jid.2014.261 . 24945094 10.1038/jid.2014.261

[37] Calvet GA, Filippis AM, Mendonca MC, Sequeira PC, Siqueira AM, Veloso VG, et al First detection of autochthonous Zika virus transmission in a HIV-infected patient in Rio de Janeiro, Brazil. J Clin Virol. 2016;74:1–3. doi: 10.1016/j.jcv.2015.11.014 . 26615388 10.1016/j.jcv.2015.11.014

[38] Penot P, Brichler S, Guilleminot J, Lascoux-Combe C, Taulera O, Gordien E, et al Infectious Zika virus in vaginal secretions from an HIV-infected woman, France, August 2016. Euro Surveill. 2017;22(3). doi: 10.2807/1560-7917.ES.2017.22.3.30444 ; PubMed Central PMCID: PMCPMC5322287. 28128730 10.2807/1560-7917.ES.2017.22.3.30444 PMC5322287

[39] Joao EC, Gouvea MI, Teixeira ML, Mendes-Silva W, Esteves JS, Santos EM, et al Zika Virus Infection Associated With Congenital Birth Defects in a HIV-infected Pregnant Woman. Pediatr Infect Dis J. 2017;36(5):500–1. doi: 10.1097/INF.0000000000001482 . 28403053 10.1097/INF.0000000000001482

[40] Pang J, Thein TL, Lye DC, Leo YS. Differential clinical outcome of dengue infection among patients with and without HIV infection: a matched case-control study. Am J Trop Med Hyg. 2015;92(6):1156–62. doi: 10.4269/ajtmh.15-0031 ; PubMed Central PMCID: PMCPMC4458819. 25825389 10.4269/ajtmh.15-0031 PMC4458819

