# Supplementary figures and images for: MAIT cells are activated in acute Dengue virus infection and after in vitro Zika virus infection
Source: PLoS Negl Trop Dis. 2018 Jan 22;12(1):e0006154. doi: 10.1371/journal.pntd.0006154 (PMC5794195; doi:10.1371/journal.pntd.0006154)

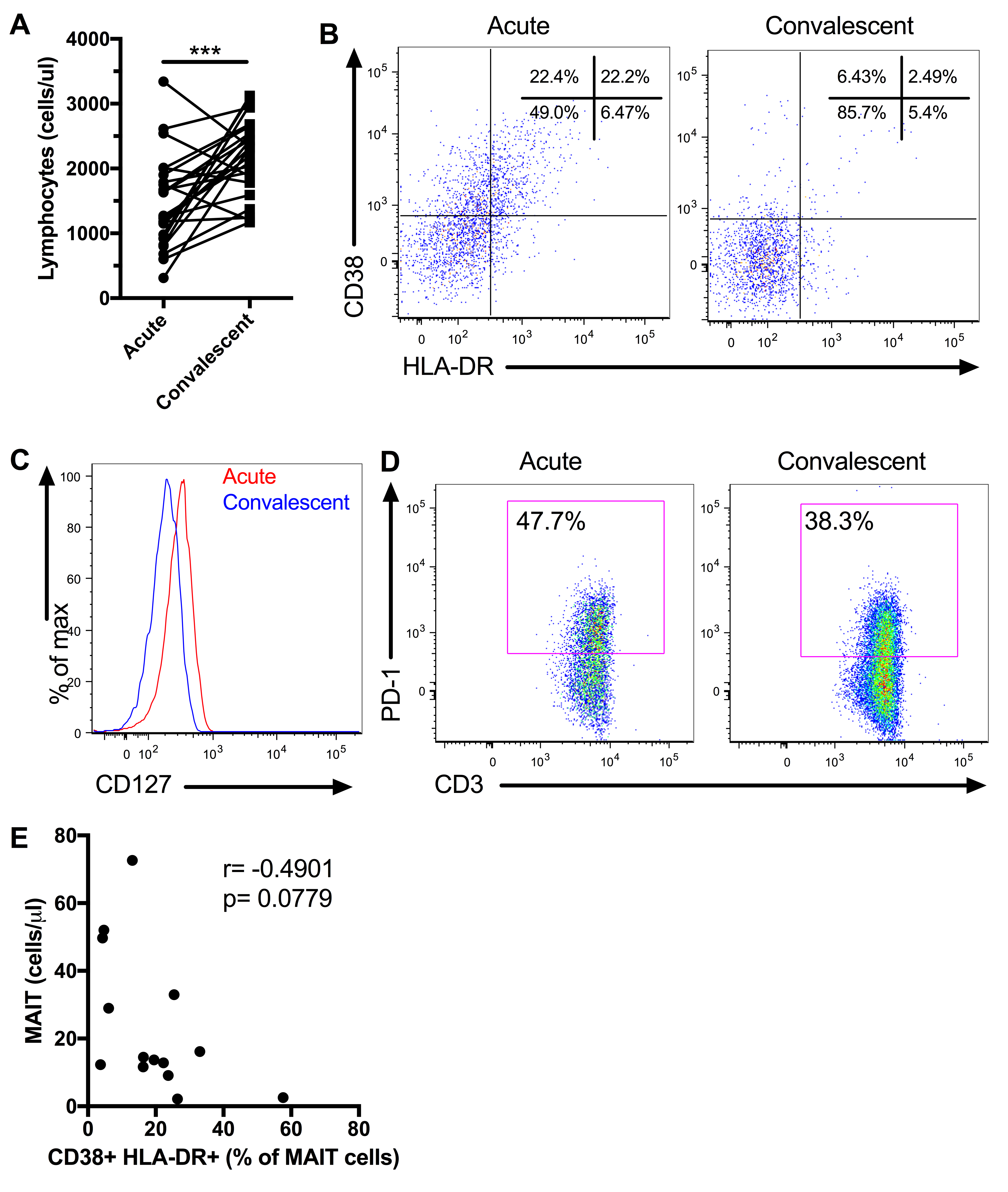

Supplement: S1 Fig — Lymphocytes count in acute and convalescent DENV infection (n = 24, A). Representative flow plots of CD38 and HLA-DR co-expression by MAIT cells in acute and convalescent DENV infection (B). CD127 expression level by MAIT cells in acute and convalescent DENV infection (C). Representative flow plots of PD-1 expression by MAIT cells in acute and convalescent DENV infection (D). Association between CD38 and HLA-DR co-expression and MAIT cells count in acute DENV infection (E). (TIFF) [file pntd.0006154.s001.tiff]

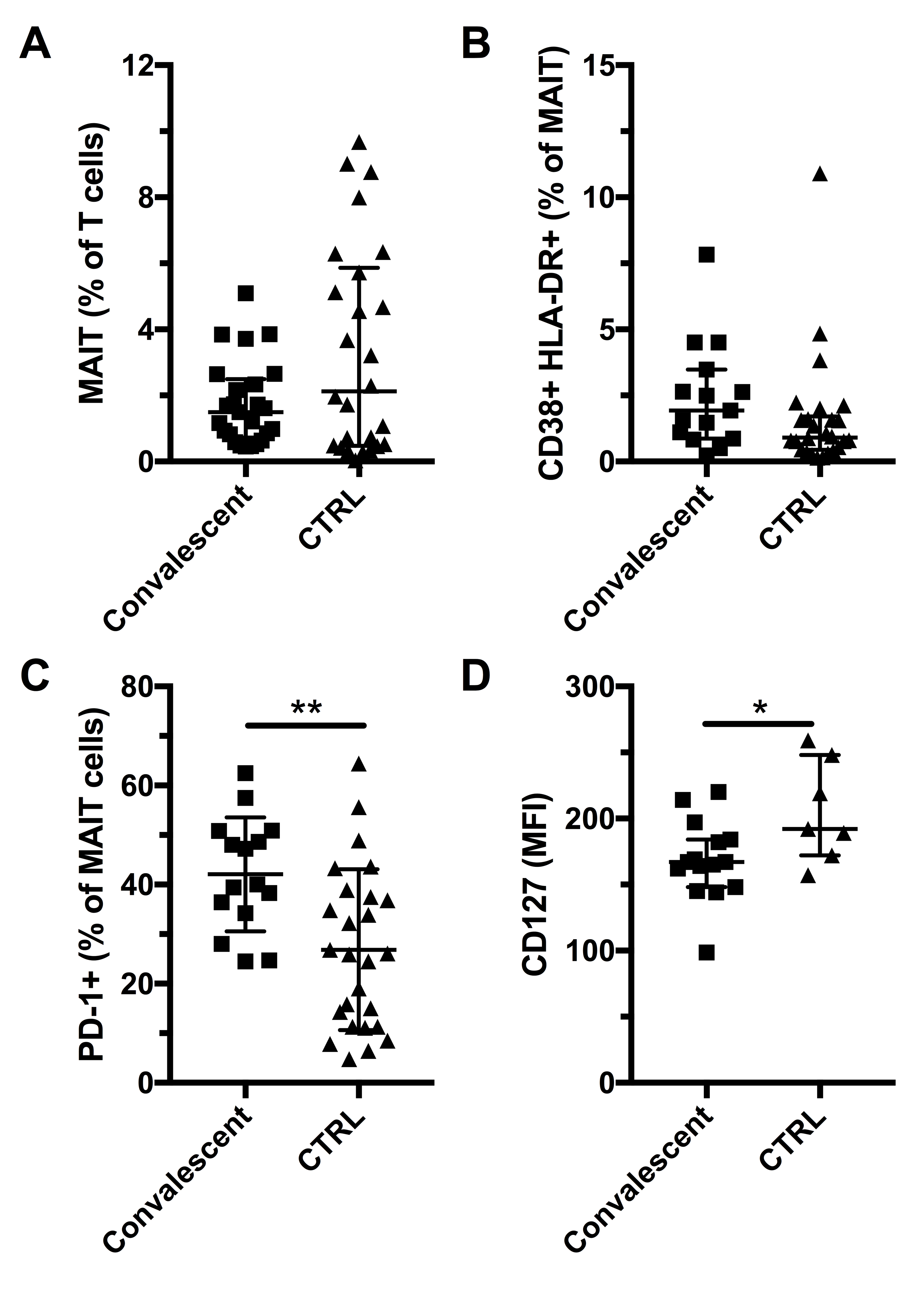

Supplement: S2 Fig — MAIT cell frequency in convalescent DENV infection (n = 25) and healthy controls (n = 26), (A). Co-expression of CD38 and HLA-DR by MAIT cells in convalescent DENV infection (n = 15) and healthy controls (n = 26), (B). PD-1 expression by MAIT cells in convalescent DENV infection (n = 15) and healthy controls (n = 26), (C). CD127 expression (MFI) by MAIT cells in convalescent DENV infection (n = 15) and healthy controls (n = 7), (D). * indicates p < 0.05, and ** indicates p < 0.01. (TIFF) [file pntd.0006154.s002.tiff]

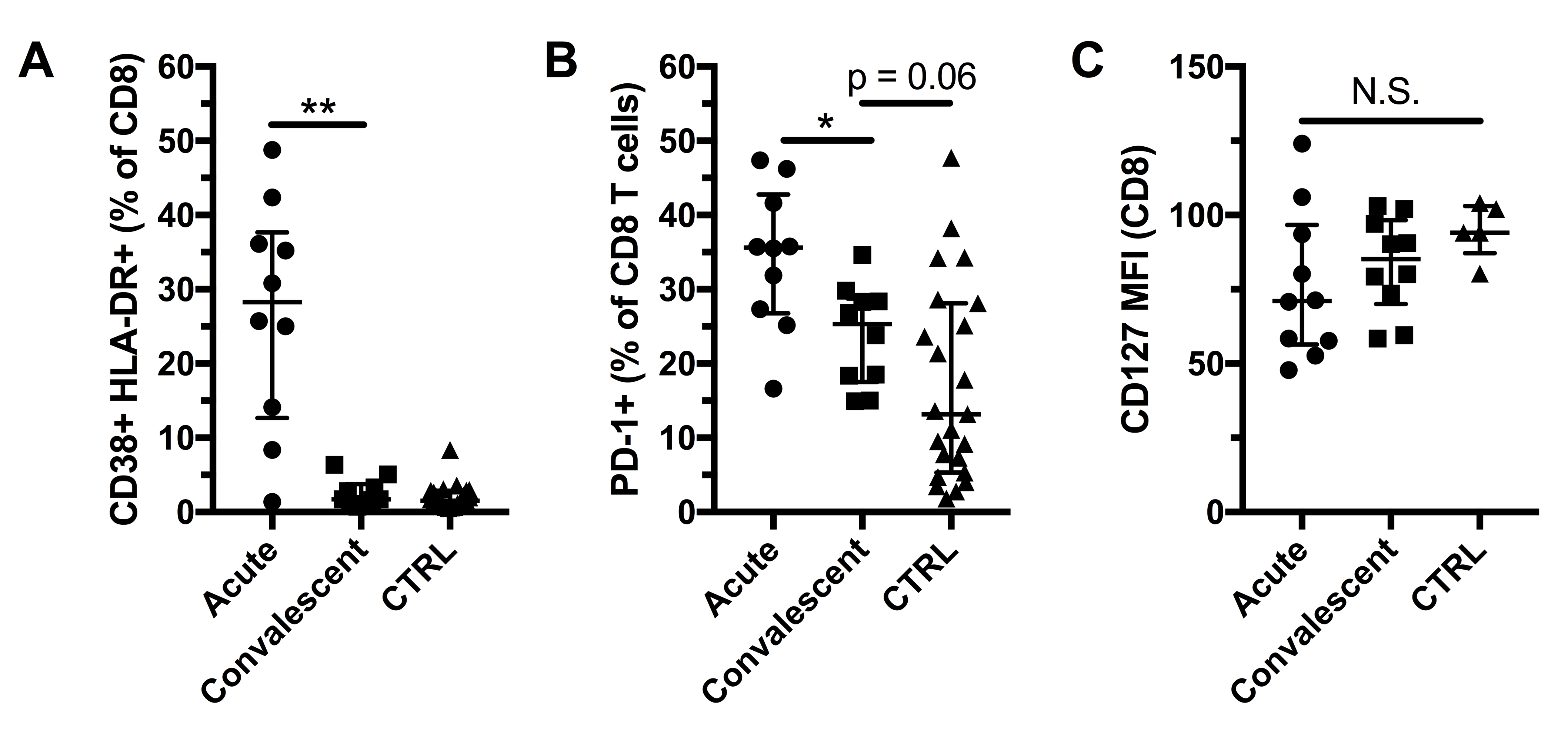

Supplement: S3 Fig — Co-expression of CD38 and HLA-DR by conventional CD8 T cells (excluding MAIT cells) during acute and convalescent DENV infection (n = 10) and healthy controls (n = 23) (A). PD-1 expression by conventional CD8 T cells in acute and convalescent DENV infection (n = 10) and healthy controls (n = 23) (B). CD127 expression by convetional CD8 T cells in acute and convalescent DENV infection (n = 10) and healthy controls (n = 5) (C). The bars and whiskers represent the median and interquartile range, respectively. * indicates p < 0.05 and ** indicates p < 0.01. (TIFF) [file pntd.0006154.s003.tiff]

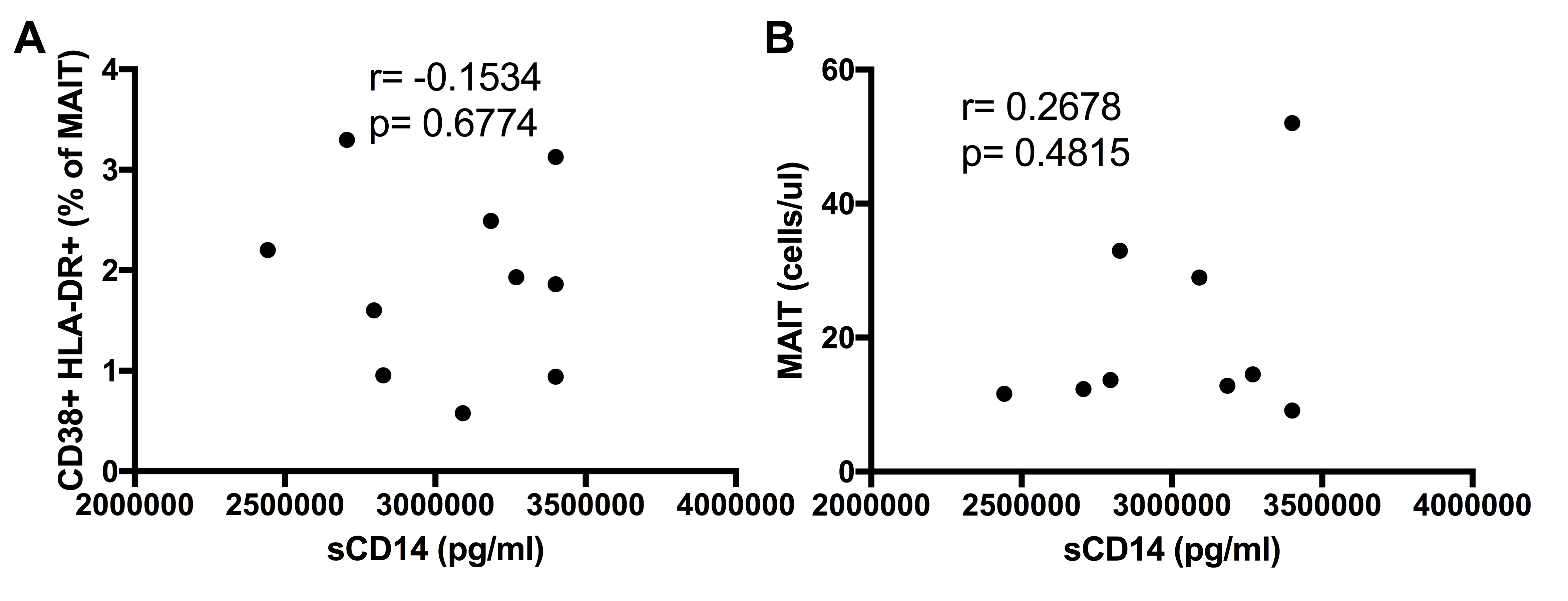

Supplement: S4 Fig — Associations between sCD14 levels and co-expression of CD38 and HLA-DR by MAIT cells (A) and MAIT cell count (B) in acute DENV infection. (TIFF) [file pntd.0006154.s004.tiff]

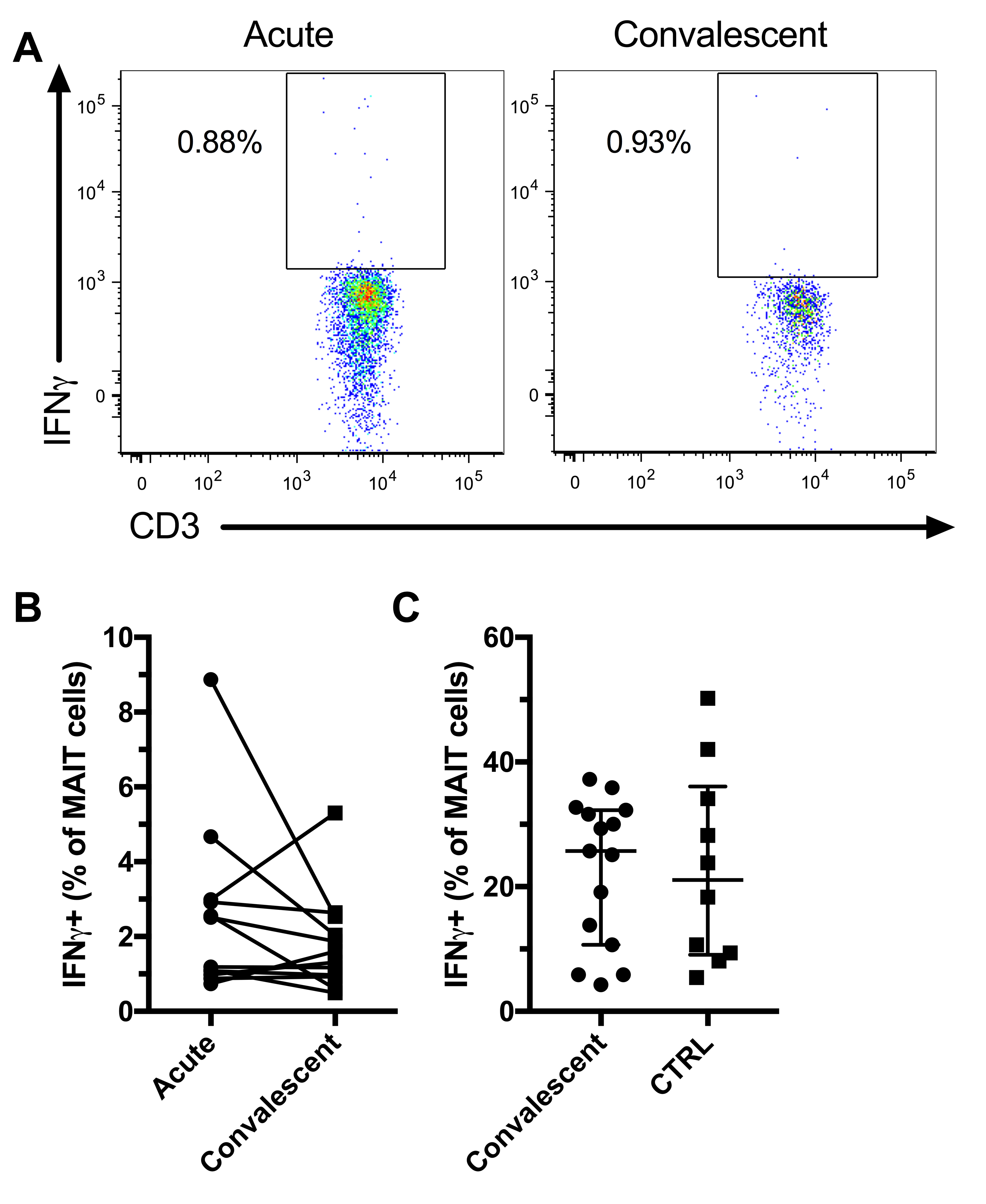

Supplement: S5 Fig — Representative flow plots showing IFNγ production by unstimulated MAIT cells in acute and convalescent dengue infection (A). IFNγ production by unstimulated MAIT cells in acute and convalescent dengue infection (n = 12, B). IFNγ production by E. coli stimulated MAIT cells in convalescent dengue infection (n = 15) and control subjects (n = 10) (C). The bars and whiskers represent the median and interquartile range, respectively. (TIFF) [file pntd.0006154.s005.tiff]
